# Kinetic Hydrate Inhibition of Natural Gels in Complex Sediment Environments

**DOI:** 10.3390/gels8120758

**Published:** 2022-11-22

**Authors:** Jianlong Wang, Jinsheng Sun, Hang Bian, Qibing Wang, Zhenbo Feng, Cheng Lu, Han Ren, Rongchao Cheng, Jintang Wang, Ren Wang

**Affiliations:** 1 School of Petroleum Engineering, China University of Petroleum (East China), Qingdao 266580, China; 2 CNPC Engineering Technology R & D Company Limited, Beijing 102206, China; 3 Center of Oil & Natural Gas Resource Exploration, China Geological Survey, Beijing 100083, China; 4 Daqing Drilling & Exploration Engineering Corporation No. 2 Drilling Company, Daqing 136413, China

**Keywords:** natural gas hydrate, sediment, natural gel, hydrate nucleation and growth, inhibition mechanism

## Abstract

Natural gels are emerging as a hotspot of global research for their greenness, environmental-friendliness, and good hydrate inhibition performance. However, previous studies mostly performed experiments for simple pure water systems and the inhibition mechanism in the sediment environment remains unclear. Given this, the inhibition performance of xanthan gum and pectin on hydrate nucleation and growth in sediment environments was evaluated via hydrate formation inhibition tests, and the inhibition internal mechanisms were revealed via a comprehensive analysis integrating various methods. Furthermore, the influences of natural gels on sediment dispersion stability and low-temperature fluid rheology were investigated. Research showed that the sediments of gas hydrate reservoirs in the South China Sea are mainly composed of micro-nano quartz and clay minerals. Xanthan gum and pectin can effectively inhibit the hydrate formation via the joint effects of the binding, disturbing, and interlayer mass transfer suppression processes. Sediments promote hydrate nucleation and yet inhibit hydrate growth. The interaction of sediments with active groups of natural gels weakens the abilities of gels to inhibit hydrate nucleation and reduce hydrate formation. Nonetheless, sediments help gels to slow down hydrate formation. Our comprehensive analysis pointed out that pectin with a concentration of 0.5 wt% can effectively inhibit the hydrate nucleation and growth while improving the dispersion stability and low-temperature rheology of sediment-containing fluids.

## 1. Introduction

Natural gas hydrates (NGHs) are clathrate solid complexes formed by water molecules and guest molecules under low temperatures and high pressure [1,2]. The total organic carbon of NGHs in the world is about twice that of conventional fossil fuels and thus, NGHs are considered an important potential high-efficiency clean alternative solution for oil and gas resources [3]. More than 90% of NGHs occur in the sea area [4,5]. During the drilling and production of marine NGHs, free gas produced via hydrate dissociation and free water of working fluids in the wellbore tends to re-form and accumulate hydrates under low temperatures and high pressure, which leads to multiple problems severely impacting the operation safety [6,7,8], such as the deterioration of the working fluid performance, blockage of blowout preventers and well oil pipelines, and other problems. Hydrate prevention has become a major challenge for the safe and efficient drilling and production of marine NGHs.

It has been proved that hydrate formation can be effectively inhibited in the presence of hydrate inhibitors in working fluids, especially kinetic hydrate inhibitors characterized by high inhibition and low dosage [9,10]. However, most typical commercial kinetic hydrate inhibitors cannot be extensively applied because of their high toxicity, high costs, and low biodegradability [11,12]. Therefore, green, environment-friendly, and biodegradable natural gels have gradually become a hot spot of research [13]. Yaqub et al. [14] reported that pectin, sodium carboxymethylcellulose, cassava starch, and xanthan gum can effectively inhibit the formation of methane hydrates; the induction time was prolonged by 47 min, 26.2 min, 37 min, and 45 min, respectively, with the additive concentration of 0.2 wt%. Wang et al. [15] found that in the case of weak driving forces for hydrate nucleation, the hydrate formation was almost completely suppressed by 0.3–0.5 wt% sodium carboxymethylcellulose. Effendi et al. [16] claimed that under the undercooling degree of 25 °C and pressure of 10 MPa, the inhibition capacity of 0.5 wt% low-methoxy pectin on the hydrate formation was triple that of PVCap.

Marine NGH-bearing sediments have abundant clay minerals. Wang et al. [6] pointed out that kaolinite can shorten the induction time of the hydrate formation; the hydrolysis of smectite interlayer cations can inhibit the hydrate formation, yet the clay structure promotes the hydrate formation. Kumar et al. [17] found that bentonite can slow down hydrate growth. Zhao et al. reported [18] that nano-scale clays reduce the induction time of the hydrate formation by 92%. Smaller particle sizes, larger specific surface areas, chemically active interfaces, and the electric charging of sediments also affect the physical and chemical properties of natural gels [19]. However, previous studies mostly evaluated the hydrate inhibition of natural gels in pure water instead of the complex system of sediment solutions. Therefore, the natural gels selected via existing research methods are probably not applicable to the drilling and production of marine NGHs.

At present, the hydrate formation inhibition of natural gels in pure water has been deeply investigated. Nonetheless, the research on hydrate prevention during the drilling and production of marine NGHs has not been publicly reported. In particular, the internal mechanism of hydrate inhibition of natural gels is still unclear. Given this, the effects of xanthan gum and pectin with 0.1–0.5 wt% concentration on hydrate formation in a 1.0 wt% sediment environment were explored via the methane hydrate inhibition evaluation test. Moreover, the Raman spectroscopy, capillary suction time test, mesostructured observation, and EDS (Energy Dispersive Spectrometry) elemental mapping were integrated to systematically reveal the internal mechanism. At last, the effects of xanthan gum and pectin on sediments were studied in view of the dispersion stability and low-temperature rheology. The research findings offer new insights into the effective applications of NGH inhibitors.

## 2. Results and Discussion

### 2.1. Basic Physical Properties of Sediments

**Figure 1:**
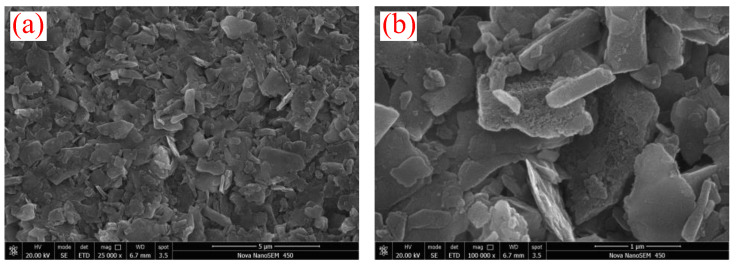
Microscopic morphology of sediments: (**a**) 5 μm; (**b**) 1 μm.

**Figure 2:**
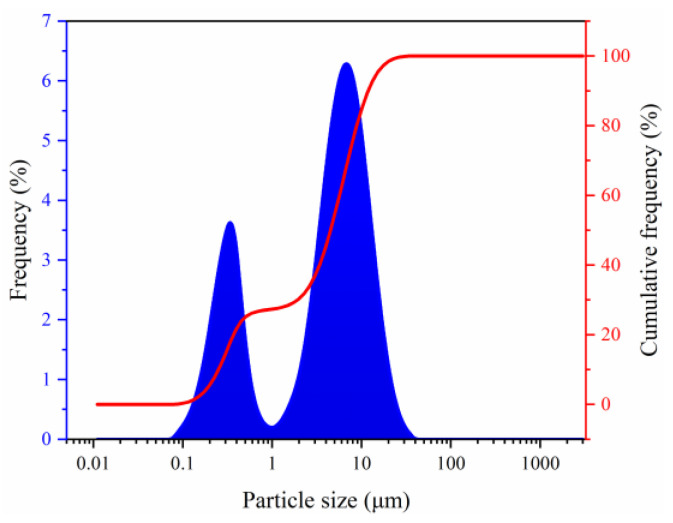
Particle size distribution of sediments.

The microscopic morphology of sediment particles was observed via the SEM and is shown in Figure 1. The sediments presented irregular laminated morphology. Moreover, the sediments were mixed with deionized water and the mixture was thoroughly stirred to test the particle size distribution. The particle size frequency curve of the sediments was bimodal (Figure 2) and the particle sizes were within 0.087–34.25 μm, with a median diameter of 4.489 μm and an average diameter of 5.336 μm, indicating micro-nano particles. Research shows that micro-nano particles affect hydrate formation. The X-ray powder diffractometer was used to analyze the bulk rock and clay mineral composition of the sediments (Table 1)—quartz and clay with the contents of 44.6 wt% and 30.8 wt%, respectively, were the main minerals of the sediments, followed by feldspar and calcite; dolomite and pyrite had relatively lower content (the total content of the two was 5.5 wt%). The clay minerals in sediments mainly included illite and illite–smectite mixed layers (41.6 wt% and 34.2 wt%, respectively), followed by kaolinite and chlorite. No smectite was observed. During drilling, the interaction between clay minerals in sediments and drilling fluids tends to trigger hydration, and the hydration degrees of clay minerals may affect the nucleation and growth of hydrates.

### 2.2. Effects of Natural Gels on Hydrate Formation

**Figure 3:**
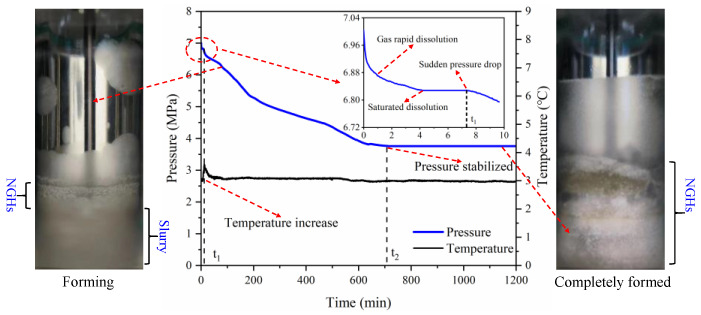
Temperature and pressure curves and experimental phenomena at key time spots during hydrate formation in 1.0 wt% sediment solutions.

The temperature and pressure curves during the hydrate formation in the 1.0 wt% sediment solution and the experimental phenomena at key time points are presented in Figure 3. The hydrate formation process is divided into three stages: gas dissolution, hydrate nucleation, and hydrate growth. When the gas dissolution reached equilibrium, the pressure in the reactor was 6.828 MPa. The occurrence of a synchronized “temperature rise” and “pressure drop” indicates the beginning of the hydrate formation. The induction time was 7.33 min. When the reaction time reached 711.58 min, the reactor pressure was stable at 3.761 MPa, which indicated that the hydrate formation in the reactor had been completed. Using the formulas in Section 2.2, the methane consumption was calculated to be 0.1662 mol and the average methane consumption rate was 2.360 × 10^−4^ mol/min. The observation via the fully transparent reactor showed hydrates were initially formed at the gas–liquid interface; with the proceeding of experiments, free water molecules in the liquid phase continuously migrated upward to the gas–liquid interface to participate in the hydrate formation, which led to the aggregation of sediment particles and finally the stratified separation between hydrates and sediments.

**Figure 4:**
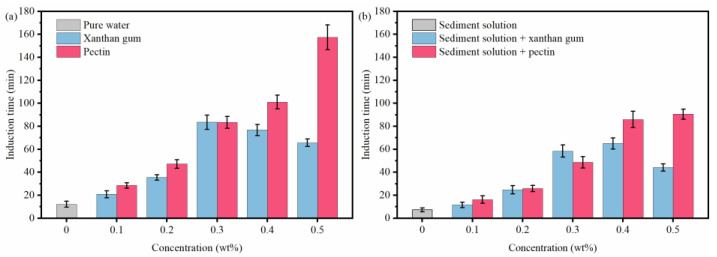
Effects of xanthan gum and pectin on the induction time of hydrate formation: (**a**) pure water systems; (**b**) sediment solution systems.

The effects of natural gels on the hydrate nucleation in pure water and sediment solutions were evaluated with respect to the induction time of each solution. As shown in Figure 4a, the induction time of hydrate formation is 12.17 min in pure water and is prolonged to a different extent with the addition of xanthan gum and pectin. This indicates that xanthan gum and pectin both have inhibitory effects on hydrate nucleation. The inhibition strength of xanthan gum first increased and then decreased with the increasing xanthan gum concentration, while that of pectin was positively correlated with its concentration. The highest induction times in the presence of xanthan gum and pectin were 65.67 min and 157.33 min, respectively. As shown in Figure 4b, the induction time of 1.0 wt% sediment solutions was 39.77% shorter than that of pure water, which means that the presence of sediments promotes the hydrate nucleation. Compared with the pure water systems with the same gel concentrations, the induction time of sediment solutions mixed with xanthan gum and pectin was also decreased, and the maximum induction times were reduced to 44.25 min and 90.58 min, respectively. This demonstrated that sediments degrade the inhibition of xanthan gum and pectin on hydrate nucleation. In addition, compared with xanthan gum, pectin still presented relatively higher inhibition on hydrate nucleation in sediment solutions.

**Figure 5:**
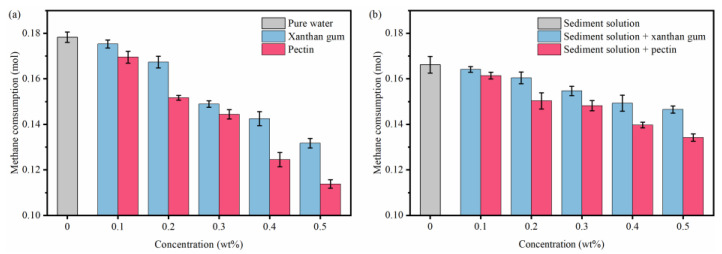
Effects of xanthan gum and pectin on the number of formed hydrates: (**a**) pure water systems; (**b**) sediment solution systems.

**Figure 6:**
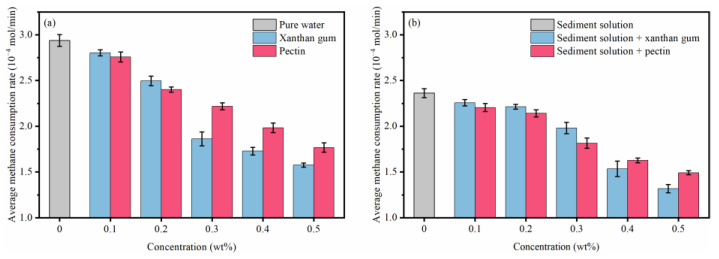
Effects of xanthan gum and pectin on the hydrate formation rate: (**a**) pure water systems; (**b**) sediment solution systems.

The effects of xanthan gum and pectin on hydrate growth were evaluated with respect to the methane consumption and average methane consumption rate during the whole experimental process. As shown in Figure 5a, 0.1783 mol of hydrates was in pure water. The addition of xanthan gum and pectin reduced the hydrate formation in solutions, and with the increasing gel concentration, formed hydrates decreased continuously. In the case of the gel concentration of 0.5 wt%, the hydrate formation in xanthan gum and pectin solutions decreased to 0.1317 mol and 0.1138 mol, respectively. Furthermore, as illustrated in Figure 5b, the hydrate formation in 1.0 wt% sediment solutions decreased by 6.742%, compared with that in pure water. With the same concentrations of xanthan gum and pectin, the gas consumption in sediment solutions was larger than that in pure water (Figure 5b), which means that more water molecules and methane molecules participated in the hydrate formation. Although the abilities of xanthan gum and pectin to reduce hydrate formation were both weakened in the sediment solutions, pectin delivered relatively stronger inhibition than that of xanthan gum. Figure 6 showed the effects of natural gels on hydrate formation rates in pure water and sediment solutions, respectively. The average methane consumption rate in pure water is 2.938 × 10^−4^ mol/min and in comparison, the presence of sediments reduces the hydrate formation rate by 19.67%. Compared with pure water systems, xanthan gum and pectin in sediment solutions can slow down the hydrate formation to a greater extent. With the gel concentration of 0.5 wt% for sediment solutions, the average methane consumption rates of xanthan gum and pectin decreased to 1.318 × 10^−4^ mol/min and 1.492 × 10^−4^ mol/min, respectively.

The above experimental results demonstrated that sediments promote the hydrate nucleation yet inhibit the hydrate growth. Moreover, they also have multiple effects on the hydrate formation inhibition of xanthan gum and pectin. To begin with, sediments weaken the hydrate nucleation inhibition of xanthan gum and pectin, which leads to a shorter induction time. Moreover, sediments degrade the performance of xanthan gum and pectin in reducing hydrate formation, which results in more formed hydrates. Finally, the hydrate formation rate in sediment solutions with xanthan gum and pectin is lower, and the presence of sediments can further enhance such deceleration of hydrate formation.

### 2.3. Analysis of Inhibition Mechanisms

**Figure 7:**
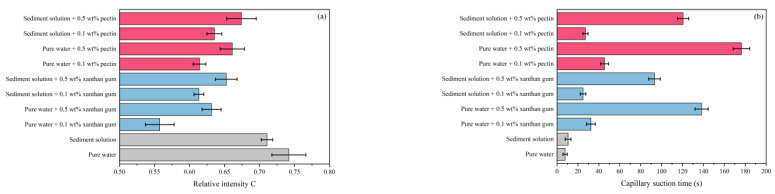
Disturbance and binding effects of natural gels on water molecules: (**a**) The relative intensity C representing the disturbance on water molecules; (**b**) Capillary suction time.

**Figure 8:**
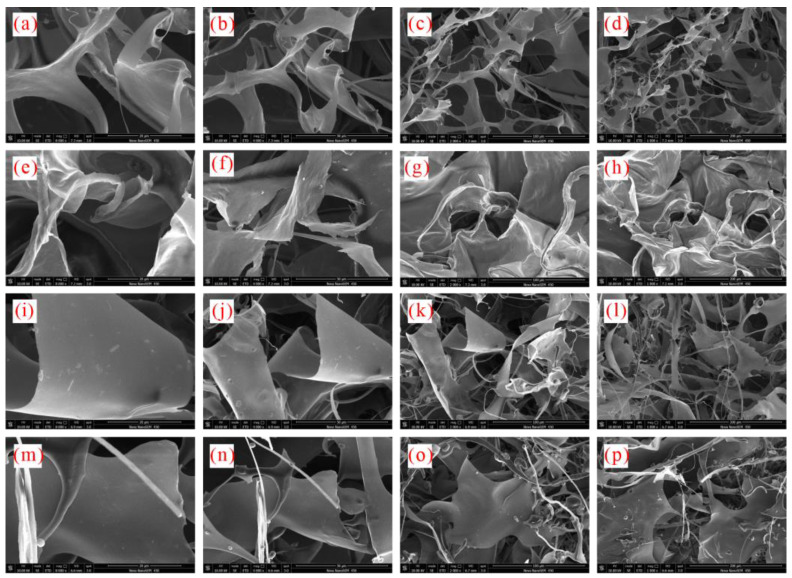
Meso-structures of lyophilized samples of xanthan gum and pectin aqueous solutions: (**a**–**d**) 0.1 wt% xanthan gum; (**e**–**h**) 0.5 wt% xanthan gum; (**i**–**l**) 0.1 wt% pectin; (**m**–**p**) 0.5 wt% pectin.

For pure water systems (Figure 7a), xanthan gum and pectin disturb water molecules. The concern is that although xanthan gum presents stronger disturbance than pectin, its ability to inhibit hydrate formation is relatively weaker, which means that the inhibition effects of xanthan gum and pectin on hydrate formation are not dependent on only their disturbance effects. Due to their hydrophilia, the active groups (–COOH and –OH) of xanthan gum and pectin can form hydrogen bonds with water molecules [20,21,22]. The capillary suction time tests (Figure 7b) proved that xanthan gum and pectin can adsorb and bind surrounding water molecules. Therefore, in the hydrate nucleation stage, xanthan gum and pectin can hinder the oriented arrangement of water molecules by binding and disturbing, and thus prevent water molecules from forming clathrate structures, which is ultimately manifested as the hydrate nucleation inhibition. Previous studies have shown that both xanthan gum and pectin can transform free water into bound water via binding, and the amount of bound water in pectin solutions is more than that in xanthan gum [23]. The bound water may not participate in the hydrate formation and thus, the formed hydrate is reduced. Figure 8 displays the mesoscopic structures of lyophilized samples of xanthan gum and pectin aqueous solutions. The xanthan gum and pectin frameworks can form network spatial structures, of which the complexity grows with the increasing gel concentration. An educated guess is that xanthan gum and pectin have interlayer mass transfer hindering effects similar to those of kinetic inhibitors such as PVP and VC-713; the polymer layer is formed on the hydrate surface by building the complex network spatial structure, which increases the mass transfer resistance and prevents water molecules from coming into contact with methane hydrate crystal nuclei. This process may be decisive, in terms of the strength of the hydrate growth inhibition. To sum up, xanthan gum and pectin inhibit hydrate growth with the comprehensive influences of binding and disturbing water molecules and interlayer mass transfer obstruction [24]. Compared with pectin, xanthan gum has a weaker binding effect, which leads to weaker hydrate nucleation inhibition. Nevertheless, it is associated with lower pore connectivity of network spatial structures and hindered free water migration. Accordingly, it delivers stronger obstruction of the interlayer mass transfer and then stronger hydrate growth inhibition.

For sediment solution systems, sediments stimulate the methane hydrate nucleation yet delay the methane hydrate growth, which is similar to the finding of REN et al. [25,26]. Sediments contain a large number of micro-nano particles, which increase the contact area between gas and liquid and also serve as the “cores” for hydrate nucleation. Moreover, due to the surface characteristics of clay minerals, clay minerals in sediments may participate in hydrate formation [27]. Consequently, sediments shorten the induction time of hydrate formation via the above processes. Wang et al. [6] found that water absorbed and bound by the hydration of clay minerals no longer participates in the hydrate formation. Therefore, the hydration of clay minerals in sediments lead to fewer formed hydrates. The laminated structure of sediments prevents water and methane molecules from migrating from the liquid phase to hydrate crystals, which increases the mass transfer resistance during hydrate formation. In addition, sediments contain abundant inorganic salt ions (such as Na^+^, Ca^2+^, and Cl^−^), which can also reduce the driving force of hydrate formation to a certain extent and slow down the hydrate formation process (Figure 1 and Figure 3).

**Figure 9:**
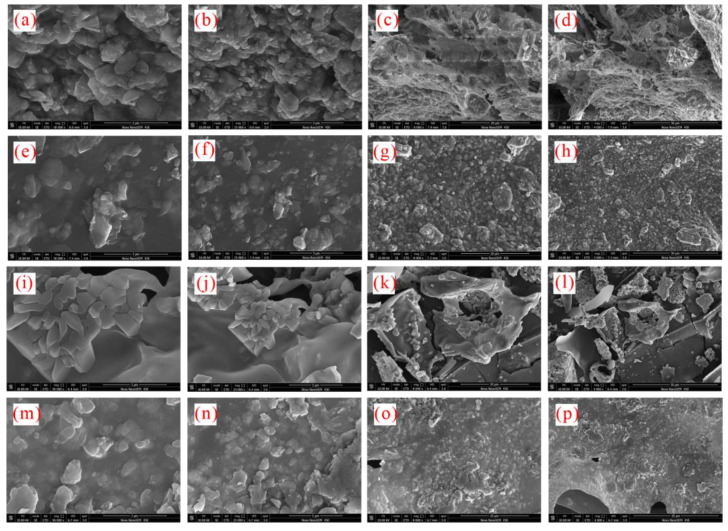
Meso-structures of lyophilized samples of xanthan gum and pectin sediment solutions: (**a**–**d**) 0.1 wt% xanthan gum; (**e**–**h**) 0.5 wt% xanthan gum; (**i**–**l**) 0.1 wt% pectin; (**m**–**p**) 0.5 wt% pectin.

**Figure 10:**
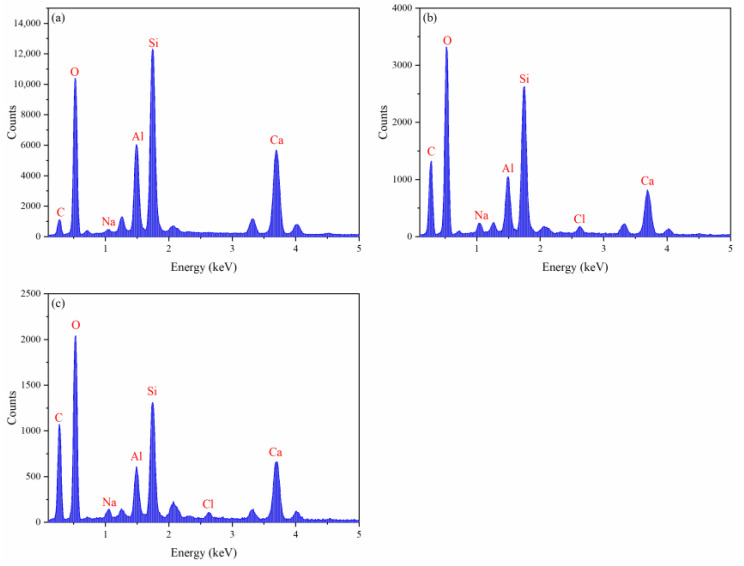
EDS elemental mapping of filter cakes of gel–sediment solutions: (**a**) sediments; (**b**) sediments + 0.5 wt% xanthan gum; (**c**) sediments + 0.5 wt% pectin.

Figure 7 reveals that the binding and disturbing effects of xanthan gum and pectin on water molecules are weakened in sediment solutions. Liu et al. claimed that kinetic inhibitors adsorbed by clay particles lose their ability to inhibit hydrate nucleation, which is likely to be the reason for the degradation of the hydrate nucleation inhibition of xanthan gum and pectin since the active groups (carbonyl and hydroxyl groups) of xanthan gum and pectin can adsorb electrically charged sediment particles by the van der Waals force or ion–dipole interaction. As shown in Figure 9, a large number of sediment particles are adsorbed on the surface of xanthan gum and pectin frameworks. To further clarify the adsorption of xanthan gum and pectin on sediments, the surface elemental distributions of filter cakes of sediment solutions before and after adding xanthan gum and pectin were measured using the EDS (Table 2 and Figure 10). The carbon (C) content increased from 10.90 wt% to 31.31 wt% and 32.96 wt%, in the cases of 1.0% sediment solutions mixed with 0.5 wt% of xanthan gum and pectin, respectively. This indicates that xanthan gum and pectin were still adsorbed onto clay mineral particles. It can be inferred that during the hydrate nucleation stage, the active groups of xanthan gum and pectin adsorb a large number of clay mineral particles, which reduces the binding and disturbing effects of gels on water molecules and thus shortens the induction time of hydrate formation; during the hydrate growth stage, more free water molecules are directionally arranged to form clathrate structures due to the decrease of the amount of bound water, which leads to more formed hydrates. The network spatial structure of xanthan gum and pectin becomes more complex and tighter and presents lower pore connectivity due to sediment adsorption (Figure 9), which results in significant growth of the resistance to the migration of water and guest molecules from the liquid phase to hydrate crystals. Such a drastic enhancement of the interlayer mass transfer suppression further reduces the hydrate formation rate.

### 2.4. Effects of Natural Gels on Sediments

**Figure 11:**
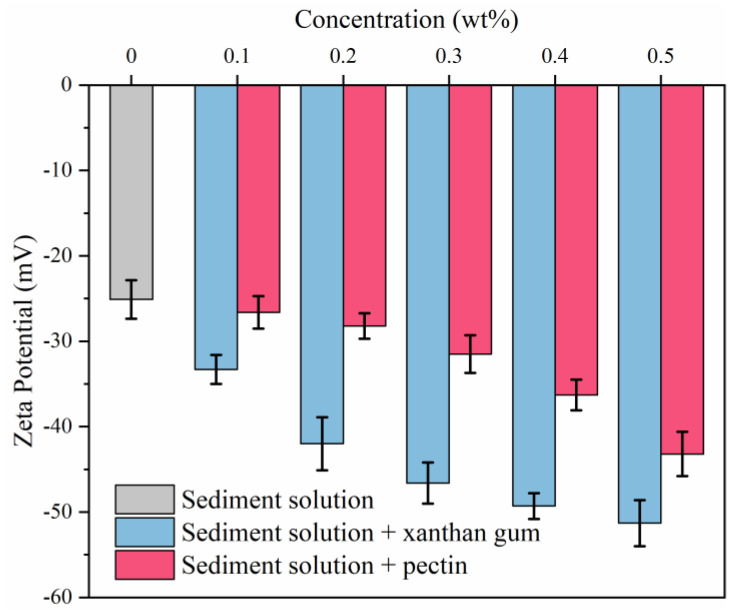
Effects of xanthan gum and pectin on the zeta potential of sediment solutions.

The zeta potential is often used to characterize the stability of drilling fluids. When the absolute value of the zeta potential is greater than 30 mV, drilling fluids are relatively stable [28,29]. As shown in Figure 11, the zeta potential of the pure sediment system is −25.1 mV, indicating inferior electrostatic repulsion and stability and high odds of settling. The zeta potential of the sediment system increases with increasing concentrations of xanthan and pectin, which means that xanthan and pectin enhance the dispersion stability of sediments in solutions. Xanthan gum and pectin contain a large number of polar groups (such as hydroxyl and carboxyl groups). The adsorption thickens the diffuse double layer of sediments and raises the electric potential. Compared with pectin, xanthan gum presented better enhancement of the dispersion stability of sediment particles; with the concentration of 0.5 wt%, the zeta potential of sediments increased to −51.3 mV.

**Figure 12:**
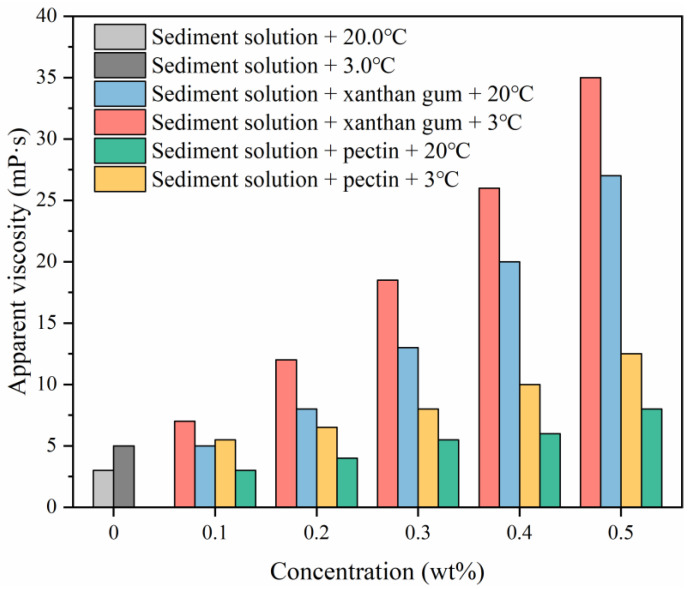
Effects of xanthan gum and pectin on the apparent viscosity of sediment solutions.

During the drilling of NGH reservoirs, the considerable change in the drilling fluid temperature greatly affects the fluid rheology and thus, it is required that drilling fluids have both high hydrate inhibition and stable rheology. This research assessed the effects of xanthan gum and pectin on the apparent viscosity of 1.0 wt% sediment solutions in the temperature range of 3–20 °C, of which the experimental results are shown in Figure 12. Clearly, the addition of xanthan gum and pectin increases the apparent viscosity of sediment solutions, yet to different degrees. The growth of apparent viscosity is positively correlated with the gel concentration. Although the apparent viscosity of sediment solutions increases slightly with a drop in temperature, sediment solutions still present high thermal stability under low temperatures. In addition, xanthan gum has been widely applied to drilling fluids as a thickening agent and delivers high viscosity growth. When the xanthan gum concentration is above 0.3 wt%, sediment solutions are excessively viscous, which tends to cause great surge pressure in the wellbore and trigger wellbore instability during drilling. The effects of pectin on the viscosity of sediment solutions are relatively small. With the pectin concentration of 0.5 wt%, the apparent viscosities of sediment solutions at 20 °C and 3 °C were 8.0 mP·s and 12.5 mP·S, respectively.

## 3. Conclusions

In this research, the inhibition performance of xanthan gum and pectin on hydrate nucleation and growth in sediments was clarified via the inhibition evaluation experiment of methane hydrate formation. Moreover, the inhibition internal mechanisms were revealed by integrating the Raman spectroscopy, capillary suction time test, mesoscopic structure observation, and EDS elemental mapping. Finally, the effects of xanthan gum and pectin on the dispersion stability of sediments and fluid rheology were studied. The following conclusions were drawn:

(1) The natural gas hydrate-bearing (NGH-bearing) sediments in the South China Sea present an irregular laminated morphology. The sediment particles are micro–nano-scale, with a median particle size of 4.489 μm. The sediments are mainly composed of quartz and clay minerals, followed by feldspar and calcite. The clay minerals mainly include illite and illite–smectite mixed layers, with the contents of 41.6 wt% and 34.2 wt%, respectively.
(2) By binding water molecules and disturbing their oriented arrangement, the resultant formation of clathrate structures, xanthan gum, and pectin prolong the induction time of hydrate nucleation. With the joint effects of the binding, disturbing, and interlayer mass transfer suppression processes, xanthan gum and pectin reduce the amount and rate of hydrate formation.
(3) Sediments promote the hydrate nucleation yet inhibit the hydrate growth. Moreover, they have multiple effects on the hydrate inhibition performance of natural gels. On the one hand, the interaction between sediments and active groups of natural gels weakens the abilities of xanthan gum and pectin to inhibit hydrate nucleation and reduce hydrate formation. On the other hand, sediments significantly enhance the interlayer mass transfer suppression of xanthan gum and pectin and thus improve their effects on slowing down hydrate formation.
(4) The comprehensive analysis pointed out the optimal gel concentration of 0.5 wt%, which delayed the induction time to 90.58 min, reduced the formed hydrates to 0.1342 mol, and decreased the hydrate formation rate to 1.492 × 10^−4^ mol/min. The hydrate formation was effectively inhibited, and the dispersion stability of sediments and the low-temperature rheology of fluids were considerably improved.

## 4. Experiments

### 4.1. Materials and Apparatus

The materials used in our experiments include methane gas (CH_4_; purity > 99.99%) supplied by Beijing Chengxin Shunxing Gas Raw Material Sales Co., Ltd. (Beijing, China); pectin (purity ≥ 98%) manufactured by Beijing MREDA Technology Co., Ltd. (Beijing, China); UPS-grade xanthan gum manufactured by Shanghai Macklin Biochemical Technology Co., Ltd. (Shanghai, China); seabed sediments collected from the South China Sea; and deionized water produced in our laboratory.

**Figure 13:**
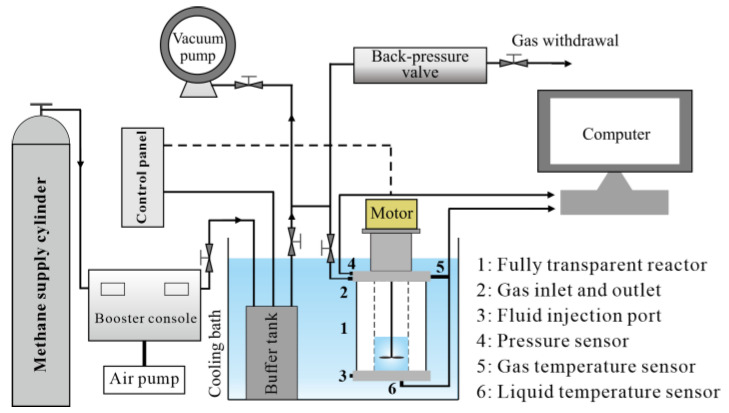
The fully transparent hydrate formation and dissociation experimental apparatus.

The hydrate formation inhibition performance was tested using a fully transparent hydrate formation and dissociation experimental apparatus (Figure 13). The fully transparent reactor had an inside diameter of 40 mm and a height of 100 mm, and the data acquisition frequency was once every 5 s. The Raman spectroscopy was performed using the LabRam HR Evolution laser confocal Raman spectrometer manufactured by Horiba (Japan-based). For the scanning electron microscopy and energy dispersive spectroscopy (SEM/EDS), the lyophilized sample solutions were first prepared with the LGJ-10D freeze dryer and then observed using the Nova nanoSEM450 field-emission scanning electron microscope (FE-SEM) manufactured by FEI (US-based).

### 4.2. Methodology

#### 4.2.1. Experimental Evaluation of Hydrate Formation Inhibition

The hydrate formation inhibition performance was tested via a constant-temperature constant-volume approach [30]. The experimental temperature was determined to be 3.0 °C, according to the mud line temperature in the Shenhu area of the South China Sea. For the purpose of comparing experimental results, the initial experimental pressure was determined to be 7.0 MPa [3]. The experimental procedure is presented below: (1) Clean the fully transparent reactor using deionized water and perform the gas tightness check of the reactor using N_2_; (2) Inject 30 mL of the sample solution into the reactor and set the stirring speed at 300 r/min and the water bath temperature at 3.0 °C; (3) After the reactor temperature is stabilized at 3.0 °C, vacuum the reactor for 30 min prior to injecting CH_4_ into the reactor until the reactor pressure reaches 7.0 MPa and then increase the stirring speed to 600 r/min; (4) Turn on the monitoring program to collect data such as temperature and pressure until the end of the experiment. In addition, the hydrate formation in the reactor during the experiment was recorded using a high-definition camera. To ensure the data reliability, each experiment was repeated three times.

The induction time, methane consumption, and average methane consumption rate are important indexes to evaluate the kinetic inhibition performance of inhibitors. The induction time is the time T_1_ characterized by the synchronized “temperature rise” and “pressure drop” in the reactor. The methane consumption represents the formed hydrate and the calculation formula is shown below [31]:

(1) $$\Delta n=\left(\frac{P_{1}}{Z_{1}}-\frac{P_{2}}{Z_{2}}\right)\times \frac{V}{\mathrm{RT}}$$

where Δn is the methane consumption, mol; R is the gas constant and equals 8.31441 J/(mol·K); T is the gas temperature, K; V is the gas volume, m^3^; and $Z_{1}$ and $Z_{2}$ are the gas compressibility factors at the beginning and end of the hydrate formation, respectively.

The average methane consumption rate is indicative of the hydrate formation rate and the calculation formula is [32]:

(2) $$\nu =\frac{\Delta n}{t_{2}-t_{1}}$$

where ν is the hydrate formation rate, mol/min; Δn is the methane consumption, mol; $t_{2}$ is the time the hydrate formation ends, min; and $t_{1}$ is the time the hydrate formation begins, min.

#### 4.2.2. Raman Spectroscopy

Measuring the disturbance degree of different components to water molecules is an important means to reveal the mechanisms of the inhibition of xanthan gum and pectin on the hydrate formation in sediments. The testing steps are as follows: (1) Take a few drops of the sample on the glass slide and cover the sample with the cover glass, place it on the microscope stage, and observe it by adjusting the stage height; (2) Excite the DPSS diode for the laser irradiation of a 532 nm green laser with the irradiation power of 50.0 mW; (3) Perform the scan within 100–4000 cm^−1^ at a time interval of 10 s and a resolution of 0.5 cm^−1^.

Usually, the relative intensity C is used as a measure of the disturbance of materials in aqueous solutions on the order degrees of water molecules [24]. C > 1 indicates that the material strengthens the ordered arrangement of water molecules, while C < 1 implies that the material disturbs the ordered arrangement of water molecules. The relative strength C is calculated below [33]:

(3) $$C=\frac{I_{L}}{I_{H}}$$

where I_L_ is the low-frequency band peak intensity (at ~3250 cm^−1^) and I_H_ is the high-frequency band peak intensity (at ~3400 cm^−1^). The peak intensity can be calculated from the peak area of the corresponding Raman peak.

#### 4.2.3. SEM/EDS Elemental Mapping

Understanding the xanthan gum, pectin, and their mesoscopic structures and surface elemental distribution in sediment solutions is helpful to gain more insights into the internal mechanism of their influence on the hydrate formation. The solution to be tested was freeze-dried, and then its mesoscopic structure was observed via the SEM. The experimental procedure followed Wang et al. EDS elemental mapping test steps are as follows: (1) Add 300 mL pure water to the three beakers, and then add 1.0 wt% sediment to each beaker. Set the stirring speed at 600 r/min, stirring for 30 min; (2) Add 0.5 wt% xanthan gum and 0.5 wt% pectin to two beakers, respectively, and continue stirring for 60 min; (3) After mixing, load the solution to be tested into the API filter press instrument and pressurize to 0.69 MPa with N_2_. The filter cake was taken out after 15 min of filtration. The relatively flat part of the mud cake was freeze-dried and then put through EDS elemental mapping.

## Figures and Tables

**Table 1:** Mineral composition of sediment.

Bulk Mineral Composition (wt%)						Clay Mineral Composition (wt%)			
Clay	Quartz	Feldspar	Calcite	Dolomite	Pyrite	Illite	I/S	Kaolinite	Chlorite
30.8	44.6	11.9	7.2	4.3	1.2	41.6	34.2	15.5	8.7

Notes: I/S represents the illite–smectite mixed layer.

**Table 2:** Elemental content of filter cakes of xanthan gum and pectin sediment solutions.

Element	Element Content (wt%)		
	Sediment	Sediment + 0.5 wt% Xanthan Gum	Sediment + 0.5 wt% Pectin
C	10.90	31.31	32.96
O	51.32	48.40	48.24
Na	0.49	1.13	0.96
Al	7.58	3.67	3.09
Si	16.25	9.63	7.26
Cl	0.00	0.55	0.47
Ca	13.46	5.31	7.02

## Data Availability

Not applicable.
